# DDT and Its Metabolites in Two Marine Dried Fish Species From Bangladesh: Residue Levels, Distribution Patterns, and Health Risk Assessment

**DOI:** 10.1002/fsn3.72286

**Published:** 2026-09-17

**Authors:** Omeo Kumar Ghosh, Md. Mazharul Islam, Fajilatun Nesa, Md. Shaikat Hasan, Ashiqul Amin Abid, Syed Akhiar Ahsan Arham, Mohammad Shoeb, Md. Iqbal Rouf Mamun

**Affiliations:** ^1^ Department of Chemistry University of Dhaka Dhaka Bangladesh; ^2^ Department of Chemistry Rajshahi University of Engineering and Technology Rajshahi Bangladesh

**Keywords:** Bangladesh, DDT levels, dried fish, food safety, GC‐ECD, health risk assessment

## Abstract

Pesticide contamination in dried fish presents a great food safety concern in Bangladesh. This study investigated the status of DDT and its metabolite levels in ninety‐six samples of two commercially available and widely consumed dried fish species, *Harpadon nehereus* and *Trichiurus lepturus* , collected across the country. The samples were extracted using the solid‐phase dispersion technique and analyzed by GC‐ECD. The analytical methodology showed excellent sensitivity and linearity with LOD ranging from 0.30 to 0.39 μg/kg, LOQ from 0.92 to 1.18 μg/kg, and regression coefficient (*R* ^*2*^) ≥ 0.9991. The parent pesticide, 4,4′‐DDT, was found in 10 out of 96 samples, with concentrations spanning from 1.41 to 11.02 μg/kg. The two metabolites, 4,4′‐DDE and 4,4′‐DDD, were more frequently detected among the analyzed specimens, with total DDTs (ΣDDTs) reaching up to 29.40 μg/kg. The contamination pattern and the proportion of 4,4′‐DDT to ΣDDTs suggested historical exposure to DDT in the food chain rather than recent application. The health risk assessment demonstrated no potential non‐carcinogenic risk, as the hazard quotient remained well below the threshold limit (< 1). The incremental lifetime cancer risk (ILCR) predominantly fell in the negligible risk range (< 1 × 10^−6^); however, a few samples (*n* = 7) showed elevated cumulative exposure (ΣILCR) but were still confined within the tolerable risk boundary (1 × 10^−6^ to 1 × 10^−4^) set by USEPA. The study provides a key insight into the current DDT contamination scenario in dried fish and aids policymakers in formulating effective and science‐driven food safety legislation in Bangladesh.

## Graphical abstract

A total of 96 dried fish samples of *Harpadon nehereus* and *Trichiurus lepturus* were analyzed by GC‐ECD to determine residues of 4,4′‐DDE, 4,4′‐DDD, and 4,4′‐DDT. The validated method showed satisfactory analytical sensitivity, with LODs of 0.30–0.39 μg/kg and LOQs of 0.92–1.18 μg/kg. The residue profile was dominated by 4,4′‐DDE, followed by 4,4′‐DDD and 4,4′‐DDT, indicating historical DDT contamination in the food chain. Only 10.42% of the samples contained detectable 4,4′‐DDT, with concentrations ranging from 1.41 to 11.02 μg/kg. Human health risk assessment showed that both ΣHQ (≤ 0.1497) and ΣILCR (≤ 2.21 × 10^−6^) were within acceptable limits, suggesting minimal health risk associated with dried fish consumption.

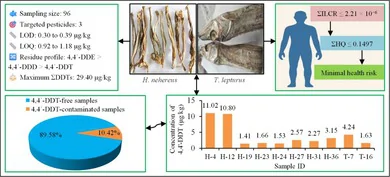

## 1 Introduction

Fish dishes are an integral part of Bengali food culture. It is an excellent source of micronutrients and health‐beneficial long‐chain polyunsaturated fatty acids (Chen et al. 2022). Aside from fresh fish, dried fish is also a very popular processed food item in Bangladesh as well as across the world (Rasul et al. 2022). In Bangladesh, dried fish acts as a substitute source of nutrients and protein for the low‐income population (Paul et al. 2018). In fact, the dried fish sector has secured a strong position in the economy of the country. After the fulfillment of local demand, a huge number of dried fish is exported all over the world each year. In the fiscal year of 2018–19, the country exported 2339.36 metric tons of dried fish, earning nearly USD 4 million, and the exported amount increased to 4141.49 metric tons in the fiscal year of 2019–20 (DoF 2020). In recent times, Bangladesh earned approximately USD 5.40 million from dried fish exports during the 2023–24 fiscal year (Dof 2024). The sun‐drying process is commonly used in the country to preserve fish, and the product is locally termed “Shutki” (Banna et al. 2022). It is a cheap and easy method for the community involved in fish drying processes. Although dried fish manufacturing processes are conducted all over the country, commercial production is limited to specific areas. An ideal place is Cox's Bazar, where fish drying is an old‐fashioned practice (Sobuj et al. 2022). Besides, the drying processes are concentrated in Khulna, Barishal, Kuakata, Sylhet, and Chalan Beel areas of Bangladesh. After large‐scale production, these dried fish are transported to several parts of the country through various market outlet systems. The drying techniques increase the shelf life of fish (Rabiepour et al. 2024), but dried fish are also susceptible to deterioration due to a variety of combined effects (Majumdar et al. 2023). In the context of Bangladesh, humid conditions, insufficient drying, inadequate hygiene, and sanitation conditions primarily promote insect infestation of dried fish (Rasul et al. 2020). Statistically, about 10%–20% of the final fish product is lost due to spoilage in Bangladesh (Khan and Khan 2001).

In response to spoilage concerns, it is conceptual that the processors and retailers use harmful organochlorine pesticides (OCPs), particularly DDT (Dichlorodiphenyltrichloroethane) in their dried fish products (Bhuiyan et al. 2008; Rasul et al. 2020; Majumdar et al. 2023). A pre‐study of the current research suggested that the people of Bangladesh are concerned about DDT contamination in dried fish, although DDT has been banned since 1997 in the country (Hasanuzzaman et al. 2018). Consequently, some people avoid dried fish products. Hence, to capture the intention of the buyers, the fish sellers often use a poster that displays “DDT‐Free Dry Fish.” The concern stemmed from the fact that few earlier studies reported the presence of DDT in dry fish samples (Siddique and Aktar 2012; Rahman et al. 2019; Kar et al. 2020; Sarker et al. 2021). Bhuiyan et al. (2009) found DDT in both the winter and rainy season variants of dry fish in the range of 3.6–250.8 and 11.1–1107.4 μg/kg, respectively. In another study, Chowdhury et al. (2010) reported high levels of total DDTs in dry fish samples ranging from 93.84 to 11,880.50 μg/kg. The origins of these residual concentrations, even after the ban, can be attributed not only to illegal pest control practices but also to the bioaccumulative and lipophilic characteristics of DDT. The pesticide possesses an immense attraction to soil and can rapidly enter water systems through runoff from contaminated regions, making it accessible to aquatic organisms like fish for bioaccumulation in fatty tissues (Ray and Shaju 2023). DDT is highly persistent in the environment, with a half‐life ranging from 4 to 30 years, and is gradually transformed into its major metabolites, DDE (Dichlorodiphenyldichloroethylene) and DDD (Dichlorodiphenyldichloroethane) (Li et al. 2010; Antu et al. 2026; ATSDR 2022). In addition to its persistence, DDT has the potential to undergo long‐range transport (Cindoruk et al. 2019), allowing its residues to be detected far from the original application sites (Li et al. 2023). However, regardless of the source, pesticides have several detrimental effects on both environmental ecology and human health (Jayaraj et al. 2016; Odewale et al. 2021). Dried fish contaminated with DDT has the potential to cause several medical ailments, including multiple types of cancer, neuropsychological dysfunction, reduced fertility, spontaneous abortion, endocrine disruption, hypothyroidism, aplastic anemia, diabetes, and developmental disorders in children (Beard 2006; Eskenazi et al. 2009; Shi et al. 2024; Mengistu et al. 2025).

A number of studies have investigated DDT residues in dried fish from Bangladesh; however, most were conducted with relatively limited sample sizes, focused on specific geographic regions and markets, or primarily emphasized the quantification of the parent DDT pesticide (Bhuiyan et al. 2008; Siddique and Aktar 2012; Hasan et al. 2014; Kar et al. 2020; Hoque et al. 2022). However, given the widespread popularity of dried fish in Bangladesh, a nationwide survey of DDT and its metabolites is essential to obtain a comprehensive understanding of contamination levels, ensure food safety, and protect public health. The current study addressed these gaps by integrating extensive nationwide sampling (96 samples from 44 sites), simultaneous determination of DDT and its major metabolites, contamination pathway analysis, and assessment of health risks from dried fish consumption. Therefore, the objective of the current study was to determine the residual concentration of 4,4′‐DDE, 4,4′‐DDD, and 4,4′‐DDT pesticides in dried fish and link their residual levels for the identification of possible contamination sources and degradation pathways. In addition, the study aimed to assess the potential non‐carcinogenic and carcinogenic risks associated with dried fish consumption using several established health risk assessment parameters and compared the results with international regulatory threshold values. The findings will provide scientific evidence necessary to understand the current contamination scenario, support the Bangladesh Food Safety Authority (BFSA) in formulating regulatory actions, and raise public awareness regarding pesticide residues in dried fish items.

## 2 Experimental

### 2.1 Study Area

**FIGURE 1:**
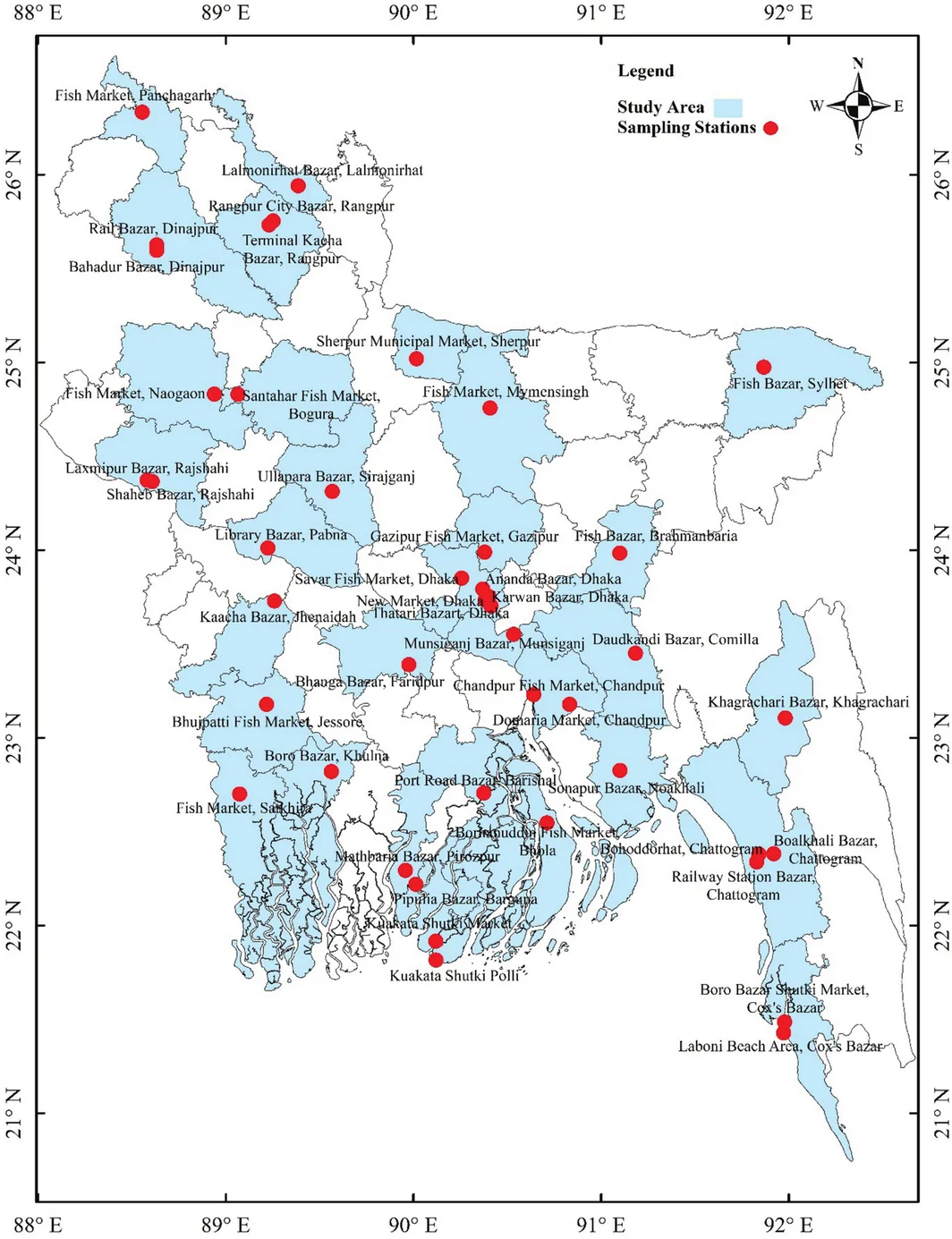
Illustration of sampling stations in a geographical map of Bangladesh.

To cover the whole of Bangladesh, the study divided the country into multiple regions and purchased dried fish samples from 44 different stations across 32 districts representing all administrative divisions of the country. The sampling locations are presented in Table 1, while their spatial distribution is illustrated in Figure 1. GPS coordinates were recorded for each of the sampling locations. These regions were selected due to their extensive fish landing activities and well‐established fish drying facilities. The residents of these regions were also found to consume dried fish dishes frequently.

**TABLE 1:** Sampling stations with corresponding GPS coordinates.

Sample ID		Sampling stations	GPS coordinates	Sample ID		Sampling stations	GPS coordinates
*H. nehereus*	*T. lepturus*			*H. nehereus*	*T. lepturus*		
H‐1	T‐1	New Market, Dhaka^a^	23.7332° N, 90.3838° E	H‐25	T‐25	Fish Bazar, Sylhet^h^	24.9742° N, 91.8679° E
H‐2	T‐2	New Market, Dhaka^a^	23.7332° N, 90.3838° E	H‐26	T‐26	Fish Bazar, Sylhet^h^	24.9742° N, 91.8679° E
H‐3	T‐3	Ananda Bazar, Dhaka^a^	23.7922° N, 90.3707° E	H‐27	T‐27	Port Road Bazar, Barishal^e^	22.7048° N, 90.3752° E
H‐4	T‐4	Thatari Bazar, Dhaka^a^	23.7013° N, 90.4144° E	H‐28	T‐28	Bhujpatti Fish Market, Jessore^d^	23.1782° N, 89.2186° E
H‐5	T‐5	Thatari Bazar, Dhaka^a^	23.7013° N, 90.4144° E	H‐29	T‐29	Boro Bazar, Khulna^d^	22.8197° N, 89.5634° E
H‐6	T‐6	Karwan Bazar, Dhaka^a^	23.7516° N, 90.4043° E	H‐30	T‐30	Fish Market, Satkhira^d^	22.7007° N, 89.0744° E
H‐7	T‐7	Karwan Bazar, Dhaka^a^	23.7516° N, 90.4043° E	H‐31	T‐31	Gazipur Fish Market, Gazipur^a^	23.9886° N, 90.3802° E
H‐8	T‐8	Savar Fish Market, Dhaka^a^	23.8505° N, 90.2580° E	H‐32	T‐32	Munsiganj Bazar, Munsiganj^a^	23.5513° N, 90.5349° E
H‐9	T‐9	Bohoddorhat, Chattogram^b^	22.3796° N, 91.8422° E	H‐33	T‐33	Sherpur Municipal Market, Sherpur^g^	25.0196° N, 90.0174° E
H‐10	T‐10	Railway Station Bazar, Chattogram^b^	22.3379° N, 91.8306° E	H‐34	T‐34	Library Bazar, Pabna^c^	24.0108° N, 89.2256° E
H‐11	T‐11	Boalkhali Bazar, Chattogram^b^	22.3819° N, 91.9208° E	H‐35	T‐35	Kaacha Bazar, Jhenaidah^d^	23.7288° N, 89.2604° E
H‐12	T‐12	Laboni Beach Area, Cox's Bazar^b^	21.4275° N, 91.9733° E	H‐36	T‐36	Domaria Market, Chandpur^b^	23.1780° N, 90.8340° E
H‐13	T‐13	Boro Bazar Shutki Market, Cox's Bazar^b^	21.4850° N, 91.9792° E	H‐37	T‐37	Chandpur Fish Market, Chandpur^b^	23.2304° N, 90.6425° E
H‐14	T‐14	Kuakata Shutki Polli, Patuakhali^e^	21.8152° N, 90.1215° E	H‐38	T‐38	Fish Bazar, Brahmanbaria^b^	23.9838° N, 91.1012° E
H‐15	T‐15	Kuakata Shutki Market, Patuakhali^e^	21.9162° N, 90.1206° E	H‐39	T‐39	Fish Market, Mymensing^g^	24.7572° N, 90.4094° E
H‐16	T‐16	Rail Bazar, Dinajpur^f^	25.6258° N, 88.6328° E	H‐40	T‐40	Pipulia Bazar, Barguna^e^	22.2191° N, 90.0138° E
H‐17	T‐17	Bahadur Bazar, Dinajpur^f^	25.5972° N, 88.6332° E	H‐41	T‐41	Mathbaria Bazar, Pirozpur^e^	22.2927° N, 89.9584° E
H‐18	T‐18	Rangpur City Bazar, Rangpur^f^	25.7542° N, 89.2533° E	H‐42	T‐42	Borhanuddin Fish Market, Bhola^e^	22.5465° N, 90.7124° E
H‐19	T‐19	Terminal Kacha Bazar, Rangpur^f^	25.7328° N, 89.2314° E	H‐43	T‐43	Sonapur Bazar, Noakhali^b^	22.8257° N, 91.1015° E
H‐20	T‐20	Shaheb Bazar, Rajshahi^c^	24.3648° N, 88.6088° E	H‐44	T‐44	Lalmonirhat Bazar, Lalmonirhat^f^	25.9411° N, 89.3871° E
H‐21	T‐21	Laxmipur Bazar, Rajshahi^c^	24.3722° N, 88.5818° E	H‐45	T‐45	Khagrachari Bazar, Khagrachari^b^	23.1054° N, 91.9828° E
H‐22	T‐22	Fish Market, Naogaon^c^	24.8314° N, 88.9405° E	H‐46	T‐46	Bhanga Bazar, Faridpur^a^	23.3895° N, 89.9768° E
H‐23	T‐23	Santahar Fish Market, Bogura^c^	24.8302° N, 89.0655° E	H‐47	T‐47	Ullapara Bazar, Sirajganj^c^	24.3119° N, 89.5691° E
H‐24	T‐24	Daudkandi Bazar, Comilla^b^	23.4501° N, 91.1846° E	H‐48	T‐48	Fish Market, Panchagarh^f^	26.3332° N, 88.5560° E

^a^ Dhaka Division.

^b^ Chattogram Division.

^c^ Rajshahi Division.

^d^ Khulna Division.

^e^ Barishal Division.

^f^ Rangpur Division.

^g^ Mymensingh Division.

^h^ Sylhet Division.

### 2.2 Sampling Strategy and Sample Pre‐Treatment

A total of 96 dried fish samples representing two species, namely *Harpadon nehereus* and *Trichiurus lepturus* , were collected with equal representation (*n* = 48) across the regions. The local names of these two varieties are Loitta ( *H. nehereus* ) and Chhuri ( *T. lepturus* ). These two species were selected because they are among the most widely marketed and popular dried marine fish species in Bangladesh, making them suitable indicators for evaluating potential dietary exposure to DDT residues (Saha et al. 2022; Begum et al. 2023). A higher number of samples were collected from Dhaka District (*n* = 16) because it is the capital city of Bangladesh and serves as the country's largest consumer market. Chattogram District (*n* = 6) was represented by the second‐highest number of samples due to its prominent role in dried fish production and trade. Four samples were collected from each of the Districts of Cox's Bazar, Patuakhali, Dinajpur, Rangpur, Rajshahi, Sylhet, and Chandpur because of their trading activity and greater product availability. Two samples (one *H. nehereus* and one *T. lepturus* ) were collected from the rest of the 23 districts.

Fish markets were selected randomly, and one representative sample was collected from each site from wholesale or retail merchants, depending on availability. Samples were collected from late May to September (monsoon season) over two consecutive years (2022 and 2023). A total of 60 samples were collected during the 2022 monsoon season and the remaining 36 samples during the 2023 monsoon season. The same sampling period was maintained in both years to minimize seasonal variability. The monsoon season was chosen because of two reasons. Firstly, consumer demand for dried fish generally increases during the monsoon season in Bangladesh when the availability of fresh fish is reduced due to unfavorable fishing conditions (Saha et al. 2022). Secondly, humidity and rainfall increase the risk of insect infestation during the storage and marketing of dried fish, potentially creating conditions in which the illegal use of DDT for pest control is more likely (Bhuiyan et al. 2009; Rasul et al. 2020). After collection, each sample was labeled with the proper labeling ID and kept in a zip‐locked bag. The samples were well preserved prior to analysis by GC‐ECD.

### 2.3 Chemical Reagents

The working organic solvents (purity 98%–99%), i.e., ethyl acetate (EtOAc), n‐hexane, and acetone, were obtained from RCI Labscan Limited, USA. Analytical reagents including anhydrous Na_2_SO_4_ (Scharlab S.L., Spain), concentrated H_2_SO_4_ (98%) (Merck, Germany), silica gel (Merck KGaA, Germany), silica sand (Kanto Chemical Co. Inc., Japan) were employed in grinding, extracting, and cleaning up phases. A composite standard mixture of organochlorine pesticides comprising 4,4′‐DDE (99%), 4,4′‐DDD (99%), and 4,4′‐DDT (98%) were purchased from Restek Corporation, Bellefonte, USA.

### 2.4 Extraction and Clean‐Up

The targeted analytes from dried fish samples were extracted by the solid‐phase dispersion technique (Islam et al. 2018), cleaned up by acid sulfonation and pipette column, and analyzed by gas chromatography equipped with electron capture detector (GC‐ECD). Initially, the collected dried fish samples were chopped into small pieces, followed by grinding into powdered form. In each case, a 10.0 g blended sample was taken in a pre‐cleaned mortar. Later, 30.0 g anhydrous Na_2_SO_4_ and 10.0 g silica sand were mixed with it properly. The powdery mixture was then successively extracted three times with 60.0, 20.0, and 20.0 mL of ethyl acetate, respectively. All the solvent extracts were combined and filtered through anhydrous Na_2_SO_4_. The clean extracts were evaporated to dryness using a rotavapor. Afterwards, the dried extracts were dissolved efficiently in 4.0 mL n‐hexane. From it, 2.0 mL was transferred quantitatively into a graduated test tube with subsequent treatment of 2.0 mL concentrated sulfuric acid saturated with n‐hexane. The treatment with concentrated sulfuric acid effectively sulfonates and destroys bulk matrix interferences such as lipids and fats, whereas chlorinated pesticides, including DDT and its metabolite, remain intact and stable (USEPA 2018; Mahugija et al. 2018; Carriera et al. 2025). Later, the test tube was carefully inverted for a minute, followed by centrifugation to separate the two layers. The upper organic phase was taken into another test tube, and the lower acidic phase was discarded. The organic phase underwent an additional clean‐up process using a silica gel–saturated sulfuric acid (2:1) packed pipette column. The utilization of a pipette column ensures the effective removal of co‐extracted interferences and matrix impurities prior to the final stage of sample preparation (Jin et al. 2022). Lastly, the clean effluent was re‐concentrated to 1.5 mL by N_2_ gas evaporator and stored in a GC vial for injecting into the gas chromatograph.

### 2.5 Analysis of Sample by GC‐ECD

The analysis of dried fish samples was carried out by gas chromatograph (GC‐2030, Shimadzu, Japan) equipped with ^63^Ni electron capture detector. The synergy of gas chromatograph and electron capture detector exhibits superior sensitivity and selectivity for halogenated pollutants like OCPs, leveraging the chlorine atom's strong affinity for electrons (Chowdhury et al. 2024; Odewale et al. 2021). The qualitative analysis of the targeted pesticides in the dried fish samples was assessed by the presence of a peak at a corresponding retention time in the sample chromatogram compared to that of the pesticide standard chromatogram. In contrast, quantitative determination of pesticide residue levels in the dried fish sample was carried out through the generation of calibration curves by using the pesticide standard solution. The Equation *y* = *mx* + *c* was used in this regard, where *y* is the peak area obtained from the chromatogram, *x* is the concentration of the final volume injected, *m* denotes the slope of the calibration curve, and *c* denotes the intercept of the calibration curve at the y‐axis. The actual concentrations of the targeted pesticides were adjusted based on the weight of the sample taken and expressed in units of μg/kg.

### 2.6 Analytical Condition of GC‐ECD

A non‐polar (HP‐5 MS) capillary column (Agilent, USA) of 30 m long × 250 μm internal diameter × 0.25 μm film thickness was used in the present study for separation purposes. Nitrogen served as both carrier gas (column flow rate: 1.92 mL/min) and make‐up gas. All the samples and standards (1.00 μL) were injected in split‐less mode. The oven temperature program was as follows: an initial temperature of 150°C (held for 2 min), followed by an increment of temperature at a rate of 5°C/min until reaching the final temperature of 295°C (held for 4 min). The injector and detector temperatures were kept at 200°C and 300°C, respectively.

### 2.7 Preparation of Pesticide Standard Solutions

The pesticide standard mixture (2000 μg/L) was initially diluted into a 50.0 μg/L solution using n‐hexane and used as a stock solution for further dilution processes. The stock solution was then serially diluted to obtain pesticide standard solutions at a concentration level of 20.0, 10.0, 5.0, 1.0, and 0.2 μg/L, respectively. After injecting the standards into GC‐ECD, calibration curves for 4,4′‐DDE, 4,4′‐DDD, and 4,4′‐DDT were developed by using the peak area of the respective concentrations in MS Excel. Representative chromatograms of target pesticide standards (Figure S1), procedural blank (Figure S2), and positive dried fish sample extracts (Figures S3–S5) are presented in Supporting Information. Peak identification was verified by comparing retention times of sample peaks with those of corresponding calibration standards under identical chromatographic conditions. A multi‐component analytical standard mixture comprising 20 Organochlorine Pesticides (OCPs) was used for calibration. However, in accordance with the specific objectives of this study, only the peaks corresponding to DDT and its metabolites (4,4′‐DDE, 4,4′‐DDD, and 4,4′‐DDT) were integrated, quantified, and reported.

### 2.8 Method Validation

Method validation is a crucial aspect of any analysis that ensures the suitability of an analytical method for its intended application. The validity of the employed method in the current research was assessed for LOD (Limit of Detection), LOQ (Limit of Quantification), and recovery percentages parameters. LOD refers to the lowest concentration of an analyte that can be detected by an analytical method, whereas LOQ denotes the lowest concentration of an analyte that can be quantified with a certain level of precision and accuracy by an analytical method. The LOD and LOQ for individual pesticides were calculated from the calibration curve based on the standard deviation of the intercept and slope of the curve (Chowdhury et al. 2024). The following Equations (1) and (2) were used for the calculation of LOD and LOQ, respectively.

(1) $$\mathrm{LOD}=\frac{3.3\times \mathrm{Std}.\;\text{deviation of intercept}\;\mathrm{at}\;y-\text{axis}}{\text{Slope of the calibration curve}}$$

(2) $$\mathrm{LOQ}=\frac{10\times \mathrm{Std}.\;\text{deviation of intercept}\;\mathrm{at}\;y-\text{axis}}{\text{Slope of the calibration curve}}$$

Sample recovery is an important method validation parameter in analytical chemistry. It refers to the proportion of an analyte that remains at the final point of determination after it undergoes multiple preparation steps, including extraction, evaporation, and purification. For the recovery experiment, a pseudo‐blank sample (H‐1) that produced signal beyond the detection limit was used as a sample matrix. A known level (2 and 5 μg/kg) of pesticide standard solution was spiked into the powdered sample matrix and allowed to stand for an hour to let the pesticides absorb into the sample. The analysis was then carried out in the same way as in the case of the sample described above. Three replications were made at each of the spiked levels, and the recovery percentages were calculated using Equation (3).

(3) $$\text{Recovery}\;\left(\%\right)=\frac{\text{Conc}.\;\text{of spiked sample}–\text{Conc}.\;\text{of blank sample}\;}{\text{Spiking concentration}}\times 100$$

### 2.9 Health Risk Assessment

Organochlorine pesticides, particularly DDT, are lipophilic and hence can build up in human fatty tissues and remain there for a prolonged time, causing major health concerns (Zhang et al. 2021). Hence, if the dried fish sample is contaminated with DDTs, it will pose a serious threat to Bangladeshi consumers. Therefore, from the aspect of food safety and public health concerns, it is essential to evaluate the health risks associated with consuming dried fish products daily. The primary method for assessing substantial risk presented to human health and ecosystems is to compare the determined concentration of a pollutant in sample with a predefined maximum residue level (Mitiku and Mitiku 2022). However, advanced risk assessment metrics such as EDI (estimated daily intake), HQ (hazard quotient), HI (hazard index), and ILCR (incremental lifetime cancer risk) are widely used to assess the potential health risks associated with exposure of pollutants through food consumption (Mengistu et al. 2025; Owagboriaye et al. 2025; Liao et al. 2024; Habibullah‐Al‐Mamun et al. 2022; Chen et al. 2020). The current study followed the combined adoption of EDI, HQ, HI, and ILCR parameters in accordance with the model supplied by the U.S. Environmental Protection Agency to establish a comprehensive framework for carcinogenic and non‐carcinogenic risk linked with dried fish consumption (USEPA 2013).

Estimated daily intake (EDI) represents the average daily dose of a chemical received per unit body weight through relevant exposure pathways and serves as a fundamental parameter in human health risk assessment. It was calculated for contaminated dried fish samples using Equation (4) (RAIS 2019; Mengistu et al. 2025).

(4) $$\mathrm{EDI}=\frac{C\times \mathrm{EF}\times \mathrm{ED}\times \mathrm{AFC}}{\mathrm{ABW}\times \mathrm{ATE}}$$

Here, *C* is the determined concentration of DDT or its metabolite in samples (μg/kg); EF refers to exposure frequency (365 days/year); ED means duration of lifetime exposure (70 years); AFC represents the estimated average consumption rate of dried fish for adults in Bangladesh (0.015 kg/person/day) (Sun et al. 2025); ABW refers to the average adults body weight in Bangladesh (60 kg/person) (Hakim et al. 2022), and ATE denotes the average exposure time (70 years × 365 days/year).

Hazard quotient (HQ) represents the ratio of the estimated daily intake of a chemical to its established safe reference dose (RfD). It signifies the possibility for non‐carcinogenic health effects posed by a single chemical and was calculated using Equation (5) (RAIS 2019; Mengistu et al. 2025).

(5) $$\mathrm{HQ}=\frac{\mathrm{EDI}}{\mathrm{RfD}}$$

Here, EDI is the estimated daily intake of micrograms of dried fish per kilogram of body weight per day (μg/kg/day), obtained from Equation (4); RfD refers to the oral reference dose (μg/kg/day) of the targeted pesticides (ATSDR 2022). If the HQ is < 1, the food is acceptable with no significant non‐carcinogenic health risk; if it is > 1, it poses a probable non‐carcinogenic risk to the consumer (USEPA 2013; Liao et al. 2024).

The HI (hazard index) refers to the cumulative health risk caused by all toxic substances encountered. In this study, it is estimated for the parent 4,4′‐DDT in combination with its two metabolites, 4,4′‐DDE and 4,4′‐DDD, using Equation (6) (RAIS 2019; Mengistu et al. 2025).

(6) $$\mathrm{HI}=\mathrm{\Sigma HQ}={\mathrm{HQ}}_{4,4\prime -\mathrm{DDE}}+{\mathrm{HQ}}_{4,4\prime -\mathrm{DDD}}+{\mathrm{HQ}}_{4,4\prime -\mathrm{DDT}}$$

This method calculates the cumulative impact of exposure to DDT and its metabolite simultaneously through dried food intake. Consumers are susceptible to a non‐carcinogenic risk if the HI is greater than one, although such a risk is less likely if the HI is less than one (USEPA 2013; Mengistu et al. 2025).

The carcinogenic risk to consumers from ingesting dried fish items was calculated using the incremental lifetime cancer risk model (ILCR). It is a risk assessment metric used to estimate the probability of an individual developing cancer over a lifetime of 70 years due to exposure to a carcinogenic substance daily. The ILCR for individual pesticides is calculated using Equation (7) (RAIS 2019; Mengistu et al. 2025).

(7) ILCR=EDI×CSF

Here, EDI (μg/kg/day) is obtained from Equation (4); CSF (kg day/μg) is the cancer slope factor that represents the upper limit likelihood of developing cancer over a lifetime exposure to a carcinogen (ATSDR 2022; Mengistu et al. 2025). The cumulative cancer risk (ΣILCR), commonly used to account for the total risk from exposure to multiple carcinogenic substances, was calculated using Equation (8) (Chen et al. 2020).

(8) $$\text{ΣILCR}={\text{ILCR}}_{4,4\prime -\mathrm{DDE}}+{\text{ILCR}}_{4,4\prime -\mathrm{DDD}}+{\text{ILCR}}_{4,4\prime -\mathrm{DDT}}$$

The ILCR range is typically categorized as no risk (< 1 × 10^−6^), acceptable risk (1 × 10^−6^ to 1 × 10^−4^), and high risk (> 1 × 10^−4^) (USEPA 2013; Mengistu et al. 2025; Haider et al. 2025). The toxicological parameter (RfD and CSF) for DDT and its metabolite is presented in Table 2 (ATSDR 2022).

**TABLE 2:** Toxicological parameters for the targeted pesticides.

Pesticides	Risk assessment parameters	
	RfD (μg/kg/day)	CSF (kg day/μg)
4,4′‐DDE	3.0 × 10^−1^	3.4 × 10^−4^
4,4′‐DDD	3.0 × 10^−2^	2.4 × 10^−4^
4,4′‐DDT	5.0 × 10^−1^	3.4 × 10^−4^

Abbreviations: CSF, cancer slope factor; RfD, reference dose.

### 2.10 Statistical Analysis

Statistical analyses were performed using SPSS software (IBM Corporation, USA). The normality of DDTs concentrations was assessed using the Shapiro–Wilk test. The distribution of pesticide levels was found to be a non‐normal one; thereby, non‐parametric statistical tests were used for subsequent statistical analyses. The discrepancies between *H. nehereus* and *T. lepturus* were evaluated using the Mann–Whitney *U* test. To address the significant difference between two major metabolites (4,4′‐DDD and 4,4′‐DDE), the Wilcoxon signed‐rank test was performed. Regional variation in ΣDDTs concentrations was evaluated by grouping the sampling districts into the eight administrative divisions of Bangladesh: Dhaka (*n* = 22), Chattogram (*n* = 22), Rajshahi (*n* = 12), Khulna (*n* = 8), Barishal (*n* = 12), Rangpur (*n* = 12), Mymensingh (*n* = 4), and Sylhet (*n* = 4). The grouped data were then analyzed for regional significance using the Kruskal–Wallis test, followed by Dunn–Bonferroni post hoc pairwise comparisons. All the differences were considered statistically significant at *p* < 0.05.

## 3 Results and Discussion

### 3.1 Method Validation

The reliability and accuracy of the analytical method employed in this study were validated through the determination of the limit of detection (LOD), limit of quantification (LOQ), and recovery efficiency. These validation parameters are summarized in Table 3. The low LOD and LOQ values reflect the high sensitivity of the method, confirming its capability to detect and quantify trace levels of DDT and its metabolite in dried fish samples. Therefore, it strengthens the method's suitability for routine monitoring of food items. The method exhibited excellent linearity over the concentration range of 0.2–20 μg/L, with a regression coefficient (*R* ^*2*^) ≥ 0.9991. Furthermore, recovery experiments were carried out to evaluate the precision and accuracy of the extraction and analytical procedures. The recovery percentages for all three compounds (4,4′‐DDE, 4,4′‐DDD, and 4,4′‐DDT) were found to be within the acceptable recovery range of 70%–120% (SANTE 2021; Perumal et al. 2025). This indicates that no significant loss or degradation of analytes occurred during sample preparation and analysis. The consistency of recovery percentages across replicates and low variability (RSD ≤ 6.98) signify the robustness and repeatability of the analytical method. Overall, the validation results confirm that the adopted methodology is a reliable, precise, and sensitive approach for determining DDT and its metabolite residues in dried fish samples, thereby ensuring the credibility of the obtained results and their applicability in food safety assessment.

**TABLE 3:** Method validation parameters for the individual pesticides.

Pesticides	Regression line	*R* ^*2*^	LOD (μg/kg)	LOQ (μg/kg)	Recovery ± RSD (%)	
					Spiking at 2 μg/kg	Spiking at 5 μg/kg
4,4′‐DDE	*y* = 4208.9*x* + 8877.1	0.9995	0.30	0.92	85.92 ± 4.21	88.45 ± 5.10
4,4′‐DDD	*y* = 2594.9*x* + 10,912	0.9991	0.38	1.16	87.05 ± 4.24	96.76 ± 5.78
4,4′‐DDT	*y* = 3190.1*x* + 7880	0.9991	0.39	1.18	82.07 ± 6.98	83.91 ± 3.23

*Note:* The recovery was expressed as mean ± relative standard deviation (RSD).

### 3.2 DDTs Residues in Dry Fish

To make a statement on the present scenario of DDTs contamination in the dried fish sector, a total of 96 statistically representative fish samples of the two most popular varieties ( *H. nehereus* and *T. lepturus* ) were purchased from different areas of Bangladesh and analyzed by GC‐ECD. The determined residual level of DDTs in the analyzed dried fish sample is tabulated in Tables 4 and 5 for *H. nehereus* and *T. lepturus* , respectively. The concentrations of 4,4′‐DDE, 4,4′‐DDD, and 4,4′‐DDT in dry fish samples varied notably across the 96 analyzed specimens. The parent pesticide, 4,4′‐DDT, was found in only 10 out of 96 samples, indicating that 89.58% of the total analyzed samples were free from DDT contamination. Concerning those ten samples (Sample ID H‐4, H‐12, H‐19, H‐23, H‐24, H‐27, H‐31, H‐36, T‐7, and T‐16), 4,4′‐DDT was found in the range of 1.41 to 11.02 μg/kg, where the *H. nehereus* fish sample collected from Thatari Bazar, Dhaka, contained the highest levels of DDT (Sample ID H‐4). According to an earlier study, the levels of DDT in four *H. nehereus* (Bombay duck) and *T. lepturus* (Ribbon fish) samples collected from several fish markets of Dhaka and Chattogram ranged from 61.9–875.0 and 131.6–149.4 μg/kg, respectively (Bhuiyan et al. 2008). In another study, Siddique and Aktar (2012) reported the mean concentration of DDT residues in the range of 125.21–181.14 μg/kg for *H. nehereus* fish samples purchased from three different markets of Chattogram and Cox's Bazar Districts. In contrast, Hasan et al. (2014) documented substantially elevated contamination levels, with DDT concentrations reaching up to 877.82 μg/kg, while investigating DDT residues in dried fish samples collected from nine different markets of Bangladesh. These distinctions in the residue level could arise from variation in sample number, fish species, sample sites, seasonal diversity, and time frame of analysis. Notably, the DDT levels found in the current study are considerably lower than those reported in earlier studies, suggesting a declining trend in contamination, possibly due to stricter regulatory control, increased public awareness, and natural degradation processes. However, the global occurrence of DDT residues is highly variable, reflecting declining contamination in some regions and continued persistence in others. For example, a long‐term monitoring study of DDT residues in six bivalve species from the coastal waters of Zhejiang, China, documented a significant decline in DDT concentrations over the period of 2002–2024 (Yu et al. 2026). Similarly, a large‐scale survey of 300 seafood samples, including various fishery products, conducted by Kim et al. (2024) in South Korea reported 4,4′‐DDT concentrations within 10 μg/kg, which are comparable to those observed in the present study. However, markedly higher DDT concentrations (727.0–1231.7 μg/kg) were reported in two species of smoke‐dried fish from Nigeria, suggesting that significant DDT contamination remains a concern in certain regions (Ofuani and Destiny 2022). Similarly, a study from New Brunswick, Canada, found elevated ΣDDTs concentrations in Brook Trout from lakes associated with historical aerial DDT applications (1952–1968), demonstrating the long‐term persistence of legacy DDT in some aquatic ecosystems (Kurek et al. 2025). In contrast, a declining trend has been reported in some developing countries, such as Ethiopia, where 4,4′‐DDT concentrations ranged from 1.25 to 8.96 μg/kg in fish samples collected from Lake Ziway (Tarekegn et al. 2025). In Bangladesh, the present findings are consistent with the study of Kar et al. (2020), which reported similar levels of DDT residue (≤ 10.00 μg/kg) in dry fish samples collected from Cox's Bazar, Kuakata (Patuakhali), and Dubla Island (Khulna) on the Bangladesh coast. Furthermore, Hoque et al. (2022) did not find any detectable level of DDT concentration in dried fish samples (*n* = 5) purchased from Cox's Bazar, Chattogram, Bhola, Patuakhali, and Khulna Districts of Bangladesh. However, the current study includes a larger sample size (*n* = 96) and covers production, distribution, and retail points across the country, providing a broader perspective on DDT contamination in dried fish.

**TABLE 4:** Residual levels of targeted pesticides in the analyzed *H. nehereus* fish samples.

Sample ID	Concentration of pesticides (μg/kg)				
	4,4′‐DDE	4,4′‐DDD	4,4′‐DDT	ΣDDTs	$\frac{4,4^{\prime }-\mathrm{DDT}}{\text{ΣDDTs}}$
H‐1	ND	ND	ND	ND	—
H‐2	ND	ND	ND	ND	—
H‐3	ND	ND	ND	ND	—
H‐4	ND	ND	11.02	11.02	1.00
H‐5	BQL	ND	BQL	BQL	—
H‐6	11.69	ND	ND	11.69	—
H‐7	2.22	ND	ND	2.22	—
H‐8	BQL	ND	ND	BQL	—
H‐9	ND	ND	ND	ND	—
H‐10	1.37	ND	ND	1.37	—
H‐11	ND	ND	ND	ND	—
H‐12	1.49	ND	10.80	12.29	0.88
H‐13	BQL	ND	ND	BQL	—
H‐14	16.63	ND	ND	16.63	—
H‐15	5.04	ND	ND	5.04	—
H‐16	BQL	ND	ND	BQL	—
H‐17	1.73	ND	ND	1.73	—
H‐18	ND	ND	BQL	BQL	—
H‐19	ND	1.59	1.41	3.00	0.47
H‐20	3.05	BQL	ND	3.05	—
H‐21	1.56	ND	ND	1.56	—
H‐22	BQL	BQL	ND	BQL	—
H‐23	5.47	3.88	1.66	11.01	0.15
H‐24	BQL	BQL	1.53	1.53	1.00
H‐25	BQL	1.61	ND	1.61	—
H‐26	3.15	3.62	ND	6.77	—
H‐27	16.19	10.64	2.57	29.40	0.09
H‐28	BQL	ND	ND	BQL	—
H‐29	BQL	ND	ND	BQL	—
H‐30	1.02	ND	ND	1.02	—
H‐31	BQL	2.65	2.27	4.92	0.46
H‐32	BQL	BQL	ND	BQL	—
H‐33	2.70	2.44	BQL	5.14	—
H‐34	BQL	ND	ND	BQL	—
H‐35	BQL	2.91	BQL	2.91	—
H‐36	9.71	7.77	3.15	20.63	0.15
H‐37	BQL	ND	ND	BQL	—
H‐38	ND	ND	ND	ND	—
H‐39	7.44	6.65	ND	14.09	—
H‐40	1.58	2.77	ND	4.35	—
H‐41	BQL	ND	ND	BQL	—
H‐42	3.67	17.62	ND	21.29	—
H‐43	0.95	ND	ND	0.95	—
H‐44	2.09	3.90	ND	5.99	—
H‐45	1.14	ND	BQL	1.14	—
H‐46	1.57	ND	ND	1.57	—
H‐47	BQL	BQL	ND	BQL	—
H‐48	1.07	BQL	ND	1.07	—

Abbreviations: BQL, below quantification limit; ND, not detected.

**TABLE 5:** Residual levels of targeted pesticides in the analyzed *T. lepturus* fish samples.

Sample ID	Concentration of pesticides (μg/kg)				
	4,4′‐DDE	4,4′‐DDD	4,4′‐DDT	ΣDDTs	$\frac{4,4^{\prime }-\mathrm{DDT}}{\text{ΣDDTs}}$
T‐1	3.20	ND	ND	3.20	—
T‐2	ND	ND	BQL	BQL	—
T‐3	ND	ND	ND	ND	—
T‐4	ND	ND	ND	ND	—
T‐5	ND	ND	ND	ND	—
T‐6	11.69	ND	ND	11.69	—
T‐7	1.09	3.37	4.24	8.70	0.49
T‐8	1.55	ND	ND	1.55	—
T‐9	1.05	ND	ND	1.05	—
T‐10	1.09	ND	ND	1.09	—
T‐11	ND	ND	ND	ND	—
T‐12	ND	ND	ND	ND	—
T‐13	2.91	ND	ND	2.91	—
T‐14	BQL	ND	ND	BQL	—
T‐15	6.69	1.90	ND	8.59	—
T‐16	1.40	6.43	1.63	9.46	0.17
T‐17	4.18	3.68	ND	7.86	—
T‐18	2.42	ND	ND	2.42	—
T‐19	9.47	6.97	ND	16.44	—
T‐20	2.21	BQL	ND	2.21	—
T‐21	1.08	ND	ND	1.08	—
T‐22	2.45	1.33	ND	3.78	—
T‐23	BQL	BQL	BQL	BQL	—
T‐24	1.52	ND	ND	1.52	—
T‐25	1.68	ND	ND	1.68	—
T‐26	ND	ND	ND	ND	—
T‐27	1.38	ND	ND	1.38	—
T‐28	ND	1.19	ND	1.19	—
T‐29	2.41	1.34	BQL	3.75	—
T‐30	1.18	ND	ND	1.18	—
T‐31	BQL	ND	ND	BQL	—
T‐32	BQL	3.88	ND	3.88	—
T‐33	1.76	BQL	ND	1.76	—
T‐34	1.05	ND	ND	1.05	—
T‐35	BQL	ND	ND	BQL	—
T‐36	BQL	ND	ND	BQL	—
T‐37	BQL	ND	BQL	BQL	—
T‐38	BQL	BQL	ND	BQL	—
T‐39	ND	ND	ND	ND	—
T‐40	BQL	5.00	ND	5.00	—
T‐41	3.50	BQL	ND	3.50	—
T‐42	5.69	4.72	ND	10.41	—
T‐43	BQL	ND	ND	BQL	—
T‐44	1.40	BQL	ND	1.40	—
T‐45	BQL	ND	ND	BQL	—
T‐46	BQL	ND	ND	BQL	—
T‐47	BQL	BQL	ND	BQL	—
T‐48	BQL	BQL	ND	BQL	—

Abbreviations: BQL, below quantification limit; ND, not detected.

The major metabolites of DDT were also examined in this study, as their occurrence in dried fish remains insufficiently documented in Bangladesh (Hoque et al. 2022; Bhuiyan et al. 2009). The two metabolites, 4,4′‐DDE and 4,4′‐DDD, ranged from 0.95 to 16.63 and 1.19 to 17.62 μg/kg, respectively, among the analyzed samples. The maximum concentration of 4,4′‐DDE was found in a *H. nehereus* fish sample collected from Kuakata Shutki Polli (Sample ID H‐14), whereas the highest residue level of 4,4′‐DDD was found in a *H. nehereus* fish sample purchased from Borhanuddin Fish Market, Bhola (Sample ID H‐42). Total DDTs (ΣDDTs) concentrations in *H. nehereus* fish samples fluctuated from below quantification limit (BQL) to 29.40 μg/kg, whereas *T. lepturus* samples exhibited ΣDDTs up to 16.44 μg/kg. The highest ΣDDTs value was recorded in sample H‐27, implying elevated contamination possibly due to direct exposure to polluted water or post‐harvest application during sun drying. Moderate concentrations, ranging from 16.44 to 21.29 μg/kg, in several samples, such as H‐14, H‐36, H‐42, and T‐19, further indicated potential contamination hotspots. This irregular distribution of the targeted pesticides reflects localized contamination rather than widespread pollution. These heterogenous distribution patterns could stem from the differences in fish collection sites, drying practices, and environmental exposure levels. This observation is supported by the Kruskal–Wallis statistical analysis (Table 6), which revealed significant regional variation in ΣDDTs concentrations among the administrative divisions of Bangladesh (*p* = 0.035). Subsequent Dunn–Bonferroni post hoc analysis showed that this variation was driven solely by a significant difference between the Barishal and Chattogram Divisions (adjusted *p* = 0.021), with no significant differences among the remaining pairwise comparisons. In the regional statistics, Barishal Division, a south‐central region bordering the Bay of Bengal, exhibited the highest mean rank, indicating a comparatively greater burden of DDT residues. The elevated concentrations observed in samples H‐27, H‐14, and H‐42 were all collected from the Barishal Division of Bangladesh (Barishal, Patukhali and Bhola Districts, respectively). The relatively elevated ΣDDTs levels observed in these regions may be influenced by several environmental factors and transit pathways. Barishal is situated within the lower Ganges–Brahmaputra–Meghna (GBM) delta, a region characterized by an extensive network of interconnected rivers and estuarine channels, including the Meghna, Arial Khan, Kirtankhola, Bishkhali, and Tentulia rivers, which ultimately drain into the northern Bay of Bengal (Wilson and Goodbred 2015). Because technical chemical mixtures were heavily applied across inland agricultural floodplains preceding the official national ban, these persistent residues continue to leach into river networks during rainfall (Uddin and Jeong 2021). As this agricultural runoff flushes downstream, it deposits heavily into terminal estuarine delta networks—particularly within the Barishal Division. Supporting this environmental setting, Haider et al. (2025) reported the occurrence of organochlorine pesticides, including DDT and its metabolites, in the surface water of the Meghna River, indicating the continued persistence and downstream transport of legacy DDT residues. Because DDTs are persistent, hydrophobic compounds with a strong affinity for particulate organic matter, they tend to accumulate in bottom sediments, which serve as long‐term reservoirs of contamination (ATSDR 2022). These residues may subsequently be transferred through aquatic food webs, contributing to elevated concentrations in marine fish. Additionally, monsoonal flooding and runoff from surrounding agricultural lands may further facilitate the redistribution and accumulation of legacy DDT residues within aquatic environments (Chaudhary et al. 2026). The significant pairwise difference between the Barishal and Chattogram Divisions revealed by the Dunn–Bonferroni post hoc analysis may be attributed to differences in fish landing centers, environmental exposure, and marketing networks. Barishal serves as an important fish landing and marketing center receiving marine fish harvested from the northern Bay of Bengal, whereas Chattogram is recognized as one of the country's principal coastal fish landing and dried fish processing regions. These differences in fisheries infrastructure and fish distribution pathways, together with localized environmental conditions, may have influenced the spatial variation in ΣDDTs concentrations observed in the present study. However, no statistically pairwise significant differences were observed among the remaining administrative divisions. This finding suggested that, apart from the comparatively elevated residue burden in Barishal, ΣDDTs contamination was relatively uniform across most regions of Bangladesh. It could be explained by the fact that both *H. nehereus* and *T. lepturus* are marine species harvested mainly from the Bay of Bengal and subsequently distributed through common marketing networks before reaching local markets. Consequently, dried fish collected from different administrative divisions may have been exposed to comparable environmental exposure, resulting in non‐significant regional variation in DDTs levels. As sampling was conducted during the monsoon seasons of 2022 and 2023, the observed residue levels likely reflect the environmental conditions prevailing during these sampling periods. Monsoonal rainfall substantially alters watershed hydrodynamics by increasing river discharge, surface runoff, and downstream transport of legacy contaminants from agricultural floodplains to estuarine and coastal ecosystems. In addition, intense rainfall and flooding may remobilize legacy DDT residues previously retained in river sediments and floodplain deposits, thereby enhancing their redistribution within aquatic environments (Alfee and Bloor 2024). However, the consistent sampling period across all administrative divisions strengthens the regional comparisons presented in the current study by minimizing potential variability arising from seasonal differences in environmental conditions. Nevertheless, future investigations covering multiple seasons would provide a more comprehensive assessment of the temporal dynamics of DDT contamination in dried fish.

**TABLE 6:** Summary of inferential statistical analyses of species, metabolite, and regional variations in DDTs residues.

Evaluation target	Statistical test	Variable matrix/Comparison	*p*	Post hoc analysis	Interpretation
Inter‐species variation	Mann–Whitney *U*	4,4′‐DDE	0.969	—	Not significant
		4,4′‐DDD	0.563	—	Not significant
		4,4′‐DDT	0.047	—	Significant
		ΣDDTs	0.421	—	Not significant
Metabolites variation	Wilcoxon signed‐rank	4,4′‐DDD vs. 4,4′‐DDE	0.006	—	Significant
Regional variation	Kruskal–Wallis	ΣDDTs	0.035	Dunn–Bonferroni	Significant only between Barishal and Chattogram Divisions (adjusted *p*_adj_ = 0.021); all other pairwise comparisons were not significant.

*Note:* Significant was considered at *p* < 0.05.

**FIGURE 2:**
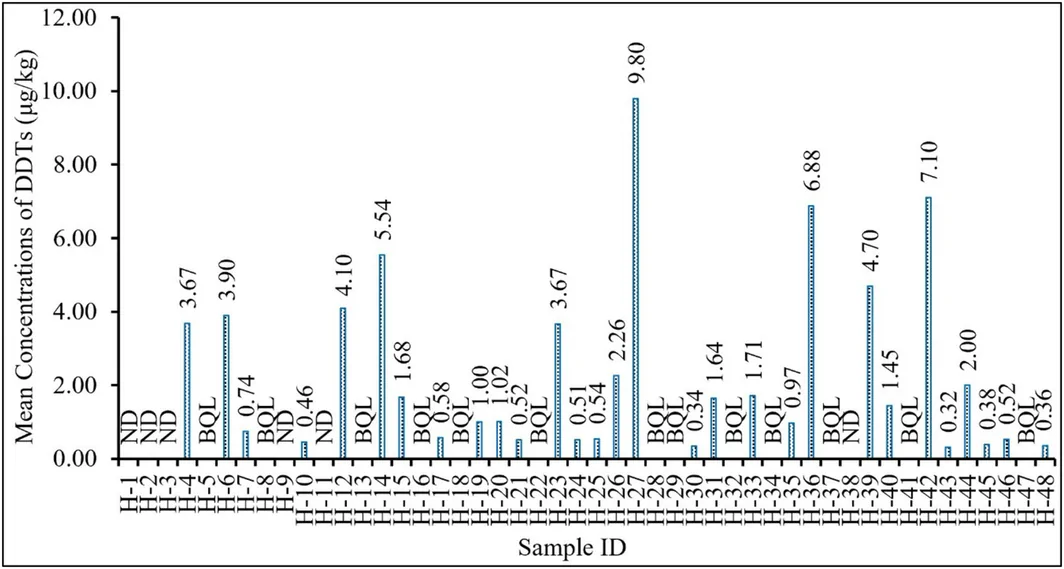
Depiction of mean concentrations of DDTs among the analyzed *H. nehereus* fish samples.

**FIGURE 3:**
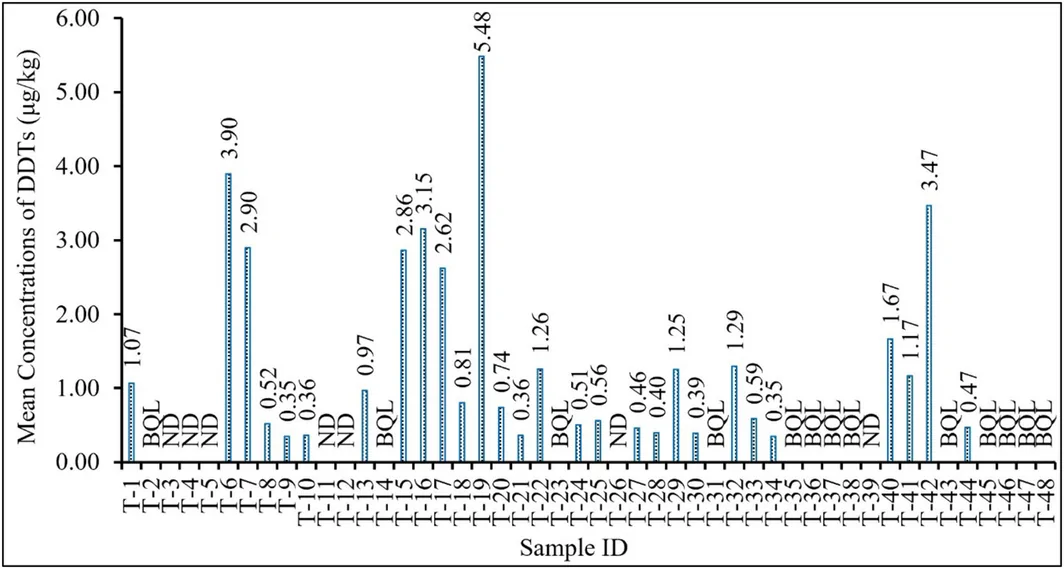
Depiction of mean concentrations of DDTs among the analyzed *T. lepturus* fish samples.

The mean concentration profile of DDTs residue in the analyzed dried fish sample is depicted in Figures 2 and 3 for *H. nehereus* and *T. lepturus* , respectively. It showed that twenty‐eight samples of *T. lepturus* and twenty‐nine samples of *H. nehereus* were contaminated with at least one of the targeted pesticides. The maximum mean concentration of DDTs residue was 5.48 μg/kg for *T. lepturus* fish species collected from Terminal Kacha Bazar, Rangpur (Sample ID T‐19). In contrast, a fish sample purchased from Port Road Bazar, Barishal (Sample ID H‐27), contained the maximum mean concentration of DDTs (9.80 μg/kg) for *H. nehereus* fish. The significance of species‐related differences in DDTs residue levels was evaluated using the Mann–Whitney *U* test (Table 6). It revealed no significant differences in the concentrations of DDE, DDD, and ΣDDTs between *H. nehereus* and *T. lepturus* (*p* > 0.05), indicating comparable overall contamination profiles between the two fish species. The absence of significant differences in DDE, DDD, and ΣDDTs concentrations between *H. nehereus* and *T. lepturus* can be attributed to uniform ecosystem exposure, as both species were collected from each of the sampling sites. In addition, both species possess comparable lipid contents, which facilitate the accumulation of lipophilic organochlorine pesticides such as DDTs, thereby contributing to similar residue burdens (Begum et al. 2023; Jamil et al. 2017). However, a marginally significant difference was observed for DDT concentrations (*p* = 0.047), with *H. nehereus* exhibiting higher mean ranks than *T. lepturus* . Yet, the rarity of DDT detection among the analyzed samples (8 samples of *H. nehereus* and 2 samples of *T. lepturus* ) suggested that this finding might not represent a consistent species‐specific contamination pattern. However, none of the samples contained any of the targeted pesticides, totally or individually, above the threshold level for fisheries items (5000 μg/kg) set by the United States Food and Drug Administration (USFDA 2000). These findings correspond with previous studies in Bangladesh reporting ΣDDTs up to 11,880.50 μg/kg in dry fish (Chowdhury et al. 2010), though the present results suggest a significant decline.

### 3.3 Correlation Among Metabolites and Contamination Pathway

**FIGURE 4:**
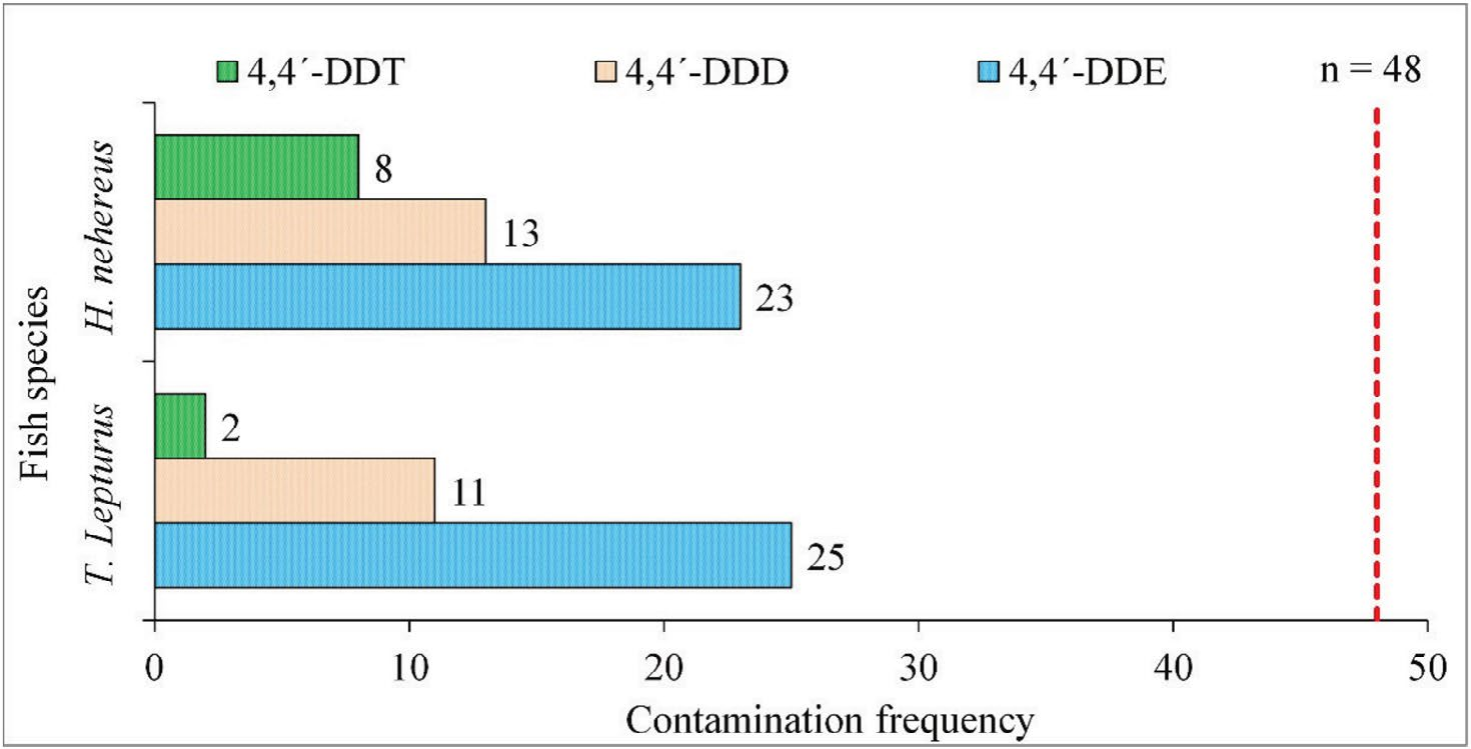
Number of dried fish samples contaminated with DDT and its metabolites in two fish varieties.

Across the fish species, the two metabolites (4,4′‐DDE and 4,4′‐DDD) were found to be present more frequently (Figure 4) and in drastically larger quantities as compared to the parent compound, 4,4′‐DDT. In Ethiopia, a study on pesticide residues in five fish species demonstrated that 4,4′‐DDE was the dominant DDT metabolite, contributing approximately 99.2% of the total DDTs concentration (Ayele et al. 2022). In the African region, a systematic review of pesticide residues on several foodstuffs, including fish products, revealed higher mean concentrations of 4,4′‐DDD compared to the parent pesticide 4,4′‐DDT (Mengistu et al. 2025). In Zimbabwe, the ratio of DDE/ΣDDT was found to be high in the sixteen fish species, indicating the dominance of metabolite products (Mhlanga and Gwisai 2024). The predominance of these metabolites is recognized as an indicator of historical exposure to DDT (Li et al. 2023). These patterns (4,4′‐DDE > 4,4′‐DDD > 4,4′‐DDT) can be attributed to the gradual transformation of 4,4′‐DDT through dehydrochlorination and reductive dechlorination processes, leading to the dominance of concentrations of 4,4′‐DDE and 4,4′‐DDD in the fish sample (ATSDR 2022; Antu et al. 2026). Consequently, strong correlations between DDT and its metabolite can infer possible common sources and degradation pathways (Wu et al. 2016). For example, the dominance of 4,4′‐DDE in most samples (e.g., H‐6, H‐14, H‐15, H‐20, T‐1, T‐6, T‐41) revealed ongoing degradation of parent DDT under aerobic environmental conditions (ATSDR 2022; Thomas et al. 2008). This oxidative transformation pathway is typical in tropical countries like Bangladesh, where intense sunlight and microbial activity accelerate the conversion of DDT to DDE. Similarly, dominance of 4,4′‐DDD in certain samples (e.g., H‐19, H‐35, T‐32, T‐40) demonstrated anaerobic degradation processes occurring either in sediment‐rich habitats or within fish tissues under low‐oxygen conditions (ATSDR 2022; Thomas et al. 2008). Statistical analysis using the Wilcoxon signed‐rank test (Table 6) showed that the residue levels of 4,4′‐DDD were significantly lower than that of 4,4′‐DDE (*p* = 0.006). Among the 96 samples, DDE exceeded DDD in 42 samples, whereas DDD exceeded DDE in only 13 samples, and equal concentrations were observed in 41 samples. The predominance of DDE over DDD indicated that aerobic degradation of DDT was more prevalent than anaerobic degradation in the regions investigated. Tarekegn et al. (2025) analyzed pesticide residues in fish samples collected from Lake Ziway, Ethiopia and found that 4,4′‐DDE represents nearly 84.97% of total DDTs, reflecting the predominance of aerobic degradation in Ethiopia. However, the concurrent detection of 4,4′‐DDE and 4,4′‐DDD in several samples, such as H‐27 (16.19 and 10.64 μg/kg) and H‐42 (3.67 and 17.62 μg/kg), suggested that both the aerobic and anaerobic degradation of DDT is occurring simultaneously in some aquatic systems. These two samples were also found to contain the highest total DDTs (ΣDDTs) concentration; however, metabolites analysis revealed that these are under sequential transformation processes. These findings align with those of Uddin et al. (2021), who also reported the co‐occurrence of DDE and DDT in dried fish collected from city markets in Dhaka. However, the degradation period of DDT can take up to 150 years in an aqueous medium (Li et al. 2023). Collectively, these observations suggested that the detected residues are more likely to originate from historical inputs and have undergone environmental degradation rather than resulting from recent uniform applications. This interpretation is consistent with the relatively low concentrations of the parent DDT compound observed in the present study, as recent widespread DDT applications would typically be expected to produce higher levels of the parent compound. Therefore, relatively low residue level implies its persistent presence in the marine environment, reflecting legacy contamination from prolonged agricultural uses, industrial discharge, and transport via surface runoff and rainfall from the application sites (Antu et al. 2026; Shaika et al. 2025). It is supported by the fact that several studies reported the presence of DDT and other pesticides in freshwater fish (Hossain et al. 2016; Khalil et al. 2021; Habibullah‐Al‐Mamun et al. 2025), as well as in the surface water of Bangladesh (Hasan et al. 2021; Chowdhury et al. 2013; Shaika et al. 2025; Haider et al. 2025). These studies indicated that legacy DDT residues continue to circulate within aquatic ecosystems and may ultimately enter marine food webs, contributing to the low but detectable residue levels observed in the present study. These are consistent with the study of Sun et al. (2020), who concluded that the DDT residues in yellowfin tuna fish of South China Sea originated from historical contamination and accumulated through biomagnification within the aquatic food web.

The ratio of parent DDT to total DDTs (DDT/ΣDDTs) is commonly used as an index to identify contamination sources, where values greater than 0.5 indicate recent input, while values below 0.5 suggest aged or historical residues (Khalil et al. 2021; Ya et al. 2019; Zamir et al. 2009). Therefore, the pollution pathway is further supported by the fact that the ratio of 4,4′‐DDT/ΣDDTs was found to be below 0.5 for most of the DDT‐positive samples (7 out of 10) in the current investigations. However, the exclusive presence of 4,4′‐DDT in the absence of its major metabolite in samples H‐4 and H‐24, along with the relatively high 4,4′‐DDT/ΣDDTs ratio observed in sample H‐12 (0.88), suggests limited degradation of the parent compound, which is indicative of a recent introduction of pesticides. Nevertheless, given the low frequency of occurrence (3 out of 96 samples) and the complexity of degradation pathways, this observation does not constitute conclusive evidence of widespread application of DDT in dried fish but rather suggests sporadic illicit usages. A similar pattern was reported by Yu et al. (2026), who found that most bivalve samples (92%) collected from coastal Zhejiang, China, reflected historical DDT contamination, whereas only a small proportion (8%) was indicative of localized recent inputs.

### 3.4 Health Risk Assessment

**FIGURE 5:**
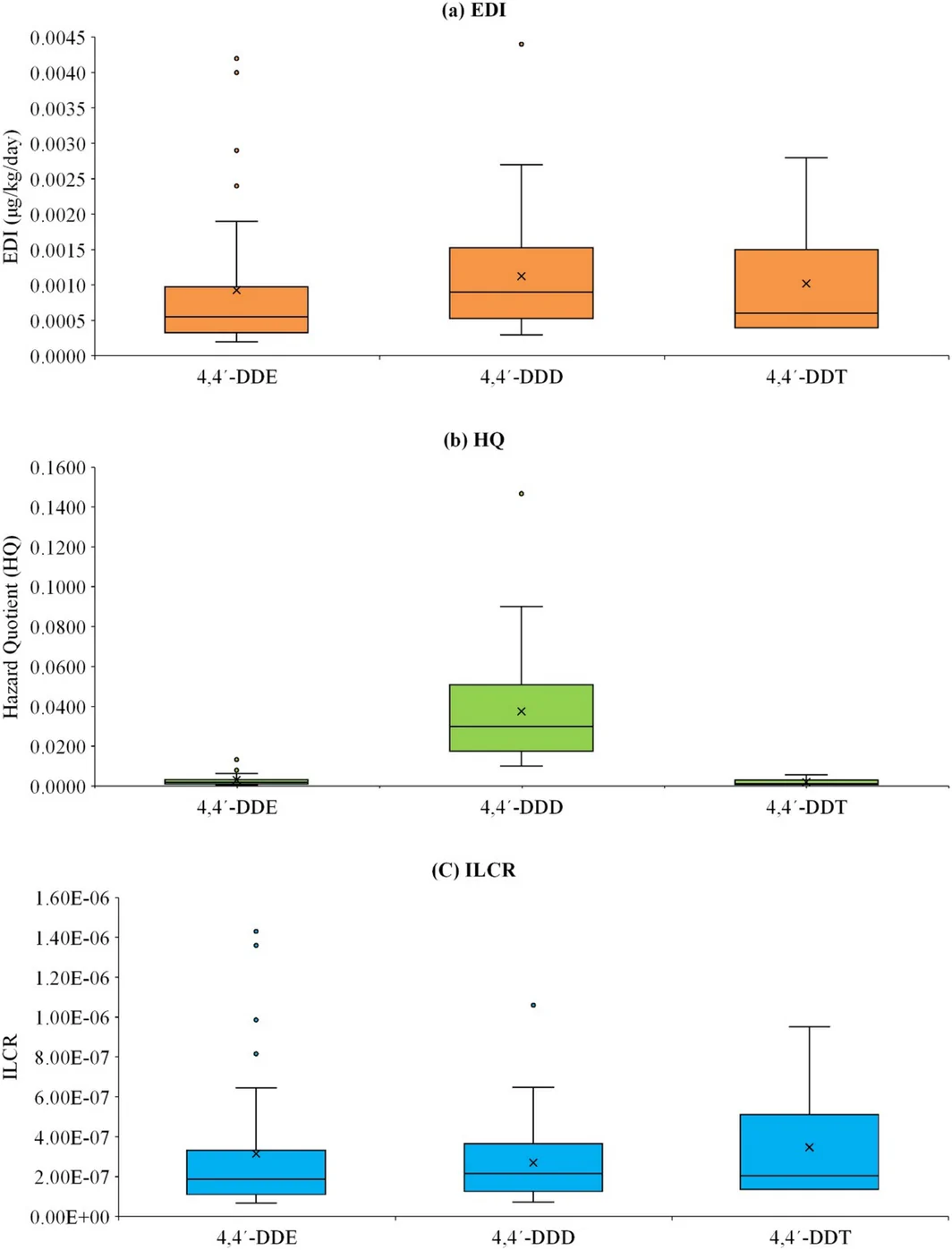
Box‐and‐whisker plots showing the distribution of health risk assessment parameters: (a) estimated daily intake (EDI), (b) hazard quotient (HQ), and (c) incremental lifetime cancer risk (ILCR).

The current study evaluated the human health risk connected with the exposure of DDT and its metabolite based on EDI (estimated daily intake), HQ (hazard quotient), HI (hazard index), and ILCR (incremental lifetime cancer risk) parameters. The findings are tabulated in Tables 7 and 8 for *H. nehereus* and *T. lepturus* , respectively. The box‐and‐whisker plots (Figure 5) illustrate the distribution of health risk parameters (EDI, HQ, and ILCR) for the dried fish samples contaminated with 4,4′‐DDE (*n* = 48), 4,4′‐DDD (*n* = 24), and 4,4′‐DDT (*n* = 10), respectively. The box indicates the interquartile range between the first quartile (Q1, 25th percentile) and the third quartile (Q3, 75th percentile), with the central line denoting the median (Q2), and the cross symbol signifying the mean value. Whiskers on both sides extended up to 1.5 times the interquartile range, and data beyond this range are shown as outlier points. The highest estimated intake was contributed by 4,4′‐DDD (0.0044 μg/kg/day), followed by 4,4′‐DDE (0.0042 μg/kg/day), and 4,4′‐DDT (0.0028 μg/kg/day). The presence of several high‐value outliers for 4,4′‐DDE indicates occasional elevated exposure to certain fish samples. In contrast, the wider interquartile range observed for 4,4′‐DDD and 4,4′‐DDT suggests greater variability in exposure levels, potentially driven by differences in residue concentrations and bioaccumulation behavior. The HQ values for all the targeted pesticides remained well below the safety threshold of 1 (USEPA 2013; Liao et al. 2024), indicating no significant non‐carcinogenic health risk associated with the consumption of dried fish. However, comparatively higher HQ values and variability were observed for 4,4′‐DDD, suggesting a relatively greater contribution of this metabolite to the overall exposure. The cumulative exposure (ΣHQ) from all three targeted pesticides was also found to be within the safety margin, with the highest value recorded for the H‐42 (0.1497), followed by the H‐27 (0.1045) sample. The incremental lifetime cancer risk (ILCR) values for the analyzed samples were predominantly within the safety limit of carcinogenic risk (< 1 × 10^−6^), apart from three samples (H‐14, H‐27, and H‐42). Notably, all three samples originated from the Barishal Division, which exhibited comparatively higher ΣDDTs residue levels in the present study. However, the ILCR values of 1.43 × 10^−6^ (4,4′‐DDE), 1.36 × 10^−6^ (4,4′‐DDE), and 1.06 × 10^−6^ (4,4′‐DDD) in samples H‐14, H‐27, and H‐42 still did not cross the acceptable carcinogenic risk regions (1 × 10^−6^—1 × 10^−4^) defined by USEPA. These findings reflect the distribution pattern in the box plot, where a few high‐value outliers were observed, signifying localization and heterogeneity in contamination patterns. The combined carcinogenic risk (ΣILCR) among the analyzed dried fish samples ranged from 6.80 × 10^−8^ to 2.21 × 10^−6^. Although a limited number of samples (*n* = 7; H‐12, H‐14, H‐27, H‐36, H‐39, H‐42, and T‐19) exceeded the negligible risk threshold of 1 × 10^−6^, none surpassed the upper acceptable limit of 1 × 10^−4^ set by USEPA. These findings are relatable to the observed EDI and HQ values, which also indicate low exposure and negligible non‐carcinogenic risk.

**TABLE 7:** Health risk assessment parameters for the contaminated *H. nehereus* fish samples.

Sample ID	EDI (μg/kg/day)			HQ				ILCR			
	4,4′‐DDE	4,4′‐DDD	4,4′‐DDT	4,4′‐DDE	4,4′‐DDD	4,4′‐DDT	ΣHQ	4,4′‐DDE	4,4′‐DDD	4,4′‐DDT	ΣILCR
H‐4	—	—	0.0028	—	—	0.0056	0.0056	—	—	9.52 × 10^−7^	9.52 × 10^−7^
H‐6	0.0029	—	—	0.0097	—	—	0.0097	9.86 × 10^−7^	—	—	9.86 × 10^−7^
H‐7	0.0006	—	—	0.0020	—	—	0.0020	2.04 × 10^−7^	—	—	2.04 × 10^−7^
H‐10	0.0003	—	—	0.0010	—	—	0.0010	1.02 × 10^−7^	—	—	1.02 × 10^−7^
H‐12	0.0004	—	0.0027	0.0013	—	0.0054	0.0067	1.36 × 10^−7^	—	9.18 × 10^−7^	1.05 × 10^−6^
H‐14	0.0042	—	—	0.0140	—	—	0.0140	1.43 × 10^−6^	—	—	1.43 × 10^−6^
H‐15	0.0013	—	—	0.0043	—	—	0.0043	4.42 × 10^−7^	—	—	4.42 × 10^−7^
H‐17	0.0004	—	—	0.0013	—	—	0.0013	1.36 × 10^−7^	—	—	1.36 × 10^−7^
H‐19	—	0.0004	0.0004	—	0.0133	0.0008	0.0141	—	9.60 × 10^−8^	1.36 × 10^−7^	2.32 × 10^−7^
H‐20	0.0008	—	—	0.0027	—	—	0.0027	2.72 × 10^−7^	—	—	2.72 × 10^−7^
H‐21	0.0004	—	—	0.0013	—	—	0.0013	1.36 × 10^−7^	—	—	1.36 × 10^−7^
H‐23	0.0014	0.0010	0.0004	0.0047	0.0333	0.0008	0.0388	4.76 × 10^−7^	2.40 × 10^−7^	1.36 × 10^−7^	8.52 × 10^−7^
H‐24	—	—	0.0004	—	—	0.0008	0.0008	—	—	1.36 × 10^−7^	1.36 × 10^−7^
H‐25	—	0.0004	—	—	0.0133	—	0.0133	—	9.60 × 10^−8^	—	9.60 × 10^−8^
H‐26	0.0008	0.0009	—	0.0027	0.0300	—	0.0327	2.72 × 10^−7^	2.16 × 10^−7^	—	4.88 × 10^−7^
H‐27	0.0040	0.0027	0.0006	0.0133	0.0900	0.0012	0.1045	1.36 × 10^−6^	6.48 × 10^−7^	2.04 × 10^−7^	2.21 × 10^−6^
H‐30	0.0003	—	—	0.0010	—	—	0.0010	1.02 × 10^−7^	—	—	1.02 × 10^−7^
H‐31	—	0.0007	0.0006	—	0.0233	0.0012	0.0245	—	1.68 × 10^−7^	2.04 × 10^−7^	3.72 × 10^−7^
H‐33	0.0007	0.0006	—	0.0023	0.0200	—	0.0223	2.38 × 10^−7^	1.44 × 10^−7^	—	3.82 × 10^−7^
H‐35	—	0.0007	—	—	0.0233	—	0.0233	—	1.68 × 10^−7^	—	1.68 × 10^−7^
H‐36	0.0024	0.0019	0.0008	0.0080	0.0633	0.0016	0.0729	8.16 × 10^−7^	4.56 × 10^−7^	2.72 × 10^−7^	1.54 × 10^−6^
H‐39	0.0019	0.0017	—	0.0063	0.0567	—	0.0630	6.46 × 10^−7^	4.08 × 10^−7^	—	1.05 × 10^−6^
H‐40	0.0004	0.0007	—	0.0013	0.0233	—	0.0246	1.36 × 10^−7^	1.68 × 10^−7^	—	3.04 × 10^−7^
H‐42	0.0009	0.0044	—	0.0030	0.1467	—	0.1497	3.06 × 10^−7^	1.06 × 10^−6^	—	1.37 × 10^−6^
H‐43	0.0002	—	—	0.0007	—	—	0.0007	6.80 × 10^−8^	—	—	6.80 × 10^−8^
H‐44	0.0005	0.0010	—	0.0017	0.0333	—	0.0350	1.70 × 10^−7^	2.40 × 10^−7^	—	4.10 × 10^−7^
H‐45	0.0003	—	—	0.0010	—	—	0.0010	1.02 × 10^−7^	—	—	1.02 × 10^−7^
H‐46	0.0004	—	—	0.0013	—	—	0.0013	1.36 × 10^−7^	—	—	1.36 × 10^−7^
H‐48	0.0003	—	—	0.0010	—	—	0.0010	1.02 × 10^−7^	—	—	1.02 × 10^−7^

Abbreviations: EDI, estimated daily intake; HQ, hazard quotient; ILCR, incremental lifetime cancer risk.

**TABLE 8:** Health risk assessment parameters for the contaminated *T. lepturus* fish samples.

Sample ID	EDI (μg/kg/day)			HQ				ILCR			
	4,4′‐DDE	4,4′‐DDD	4,4′‐DDT	4,4′‐DDE	4,4′‐DDD	4,4′‐DDT	ΣHQ	4,4′‐DDE	4,4′‐DDD	4,4′‐DDT	ΣILCR
T‐1	0.0008	—	—	0.0027	—	—	0.0027	2.72 × 10^−7^	—	—	2.72 × 10^−7^
T‐6	0.0029	—	—	0.0097	—	—	0.0097	9.86 × 10^−7^	—	—	9.86 × 10^−7^
T‐7	0.0003	0.0008	0.0011	0.0010	0.0267	0.0022	0.0299	1.02 × 10^−7^	1.92 × 10^−7^	3.74 × 10^−7^	6.68 × 10^−7^
T‐8	0.0004	—	—	0.0013	—	—	0.0013	1.36 × 10^−7^	—	—	1.36 × 10^−7^
T‐9	0.0003	—	—	0.0010	—	—	0.0010	1.02 × 10^−7^	—	—	1.02 × 10^−7^
T‐10	0.0003	—	—	0.0010	—	—	0.0010	1.02 × 10^−7^	—	—	1.02 × 10^−7^
T‐13	0.0007	—	—	0.0023	—	—	0.0023	2.38 × 10^−7^	—	—	2.38 × 10^−7^
T‐15	0.0017	0.0005	—	0.0057	0.0167	—	0.0224	5.78 × 10^−7^	1.20 × 10^−7^	—	6.98 × 10^−7^
T‐16	0.0004	0.0016	0.0004	0.0013	0.0533	0.0008	0.0554	1.36 × 10^−7^	3.84 × 10^−7^	1.36 × 10^−7^	6.56 × 10^−7^
T‐17	0.0010	0.0009	—	0.0033	0.0300	—	0.0333	3.40 × 10^−7^	2.16 × 10^−7^	—	5.56 × 10^−7^
T‐18	0.0006	—	—	0.0020	—	—	0.0020	2.04 × 10^−7^	—	—	2.04 × 10^−7^
T‐19	0.0024	0.0017	—	0.0080	0.0567	—	0.0647	8.16 × 10^−7^	4.08 × 10^−7^	—	1.22 × 10^−6^
T‐20	0.0006	—	—	0.0020	—	—	0.0020	2.04 × 10^−7^	—	—	2.04 × 10^−7^
T‐21	0.0003	—	—	0.0010	—	—	0.0010	1.02 × 10^−7^	—	—	1.02 × 10^−7^
T‐22	0.0006	0.0003	—	0.0020	0.0100	—	0.0120	2.04 × 10^−7^	7.20 × 10^−8^	—	2.76 × 10^−7^
T‐24	0.0004	—	—	0.0013	—	—	0.0013	1.36 × 10^−7^	—	—	1.36 × 10^−7^
T‐25	0.0004	—	—	0.0013	—	—	0.0013	1.36 × 10^−7^	—	—	1.36 × 10^−7^
T‐27	0.0003	—	—	0.0010	—	—	0.0010	1.02 × 10^−7^	—	—	1.02 × 10^−7^
T‐28	—	0.0003	—	—	0.0100	—	0.0100	—	7.20 × 10^−8^	—	7.20 × 10^−8^
T‐29	0.0006	0.0003	—	0.0020	0.0100	—	0.0120	2.04 × 10^−7^	7.20 × 10^−8^	—	2.76 × 10^−7^
T‐30	0.0003	—	—	0.0010	—	—	0.0010	1.02 × 10^−7^	—	—	1.02 × 10^−7^
T‐32	—	0.0010	—	—	0.0333	—	0.0333	—	2.40 × 10^−7^	—	2.40 × 10^−7^
T‐33	0.0004	—	—	0.0013	—	—	0.0013	1.36 × 10^−7^	—	—	1.36 × 10^−7^
T‐34	0.0003	—	—	0.0010	—	—	0.0010	1.02 × 10^−7^	—	—	1.02 × 10^−7^
T‐40	—	0.0013	—	—	0.0433	—	0.0433	—	3.12 × 10^−7^	—	3.12 × 10^−7^
T‐41	0.0009	—	—	0.0030	—	—	0.0030	3.06 × 10^−7^	—	—	3.06 × 10^−7^
T‐42	0.0014	0.0012	—	0.0047	0.0400	—	0.0447	4.76 × 10^−7^	2.88 × 10^−7^	—	7.64 × 10^−7^
T‐44	0.0004	—	—	0.0013	—	—	0.0013	1.36 × 10^−7^	—	—	1.36 × 10^−7^

Abbreviations: EDI, estimated daily intake; HQ, hazard quotient; ILCR, incremental lifetime cancer risk.

The health risk assessment in the present study suggested that the non‐carcinogenic and carcinogenic risks associated with the consumption of dried fish are generally within internationally accepted safety thresholds, suggesting minimal potential health risk for consumers under the current exposure scenario. The findings are consistent with Hoque et al. (2022), who concluded that the pesticide residue would not pose a potential health risk to the dried fish consumer based on the risk parameters of EDI (estimated daily intake) and HRI (health risk index). In another study, Rahman et al. (2019) calculated the risk assessment parameter for the DDT‐contaminated dried fish sample and observed that most of the samples were within the acceptable thresholds for both the non‐carcinogenic and carcinogenic risk. Earlier studies in the country have mainly relied on maximum residue levels or predefined threshold values to assess the safety of dried fish consumption (Bhuiyan et al. 2009; Siddique and Aktar 2012; Hasan et al. 2014; Kar et al. 2020). Therefore, the present study extends these investigations by additionally incorporating internationally accepted health risk assessment metrics (EDI, HQ, HI, ILCR, and ΣILCR), together with an extensive sampling campaign (*n* = 96), to provide a better understanding of consumer exposure. The present findings are also in agreement with several international studies. In China, Huang et al. (2025) observed that the carcinogenic and non‐carcinogenic risks associated with organochlorine pesticides exposure through the consumption of cultured common carp muscle is within acceptable zone. Similarly, a study from South Korea reported that the consumption of domestically farmed fish poses minimal health risk with respect to the pesticide levels (Kim et al. 2024). In contrast, a study from northern Ghana reported that although the non‐carcinogenic risk indicators (HQ and HI) were within acceptable limits, the levels of 4,4′‐DDE and 4,4′‐DDD suggested a potential carcinogenic risk from consuming fish collected from the Libga and Builpela reservoirs (Adokiya et al. 2025). Likewise, some studies from Nigeria have reported potential health risks associated with the consumption of cured fish products (Ofuani and Destiny 2022; Uzomah et al. 2021). These contrasting findings indicated that the health risks associated with DDT exposure through fish consumption vary geographically, depending on contamination levels, environmental persistence, and local dietary exposure patterns.

### 3.5 Limitation of the Study

In the present study, DDT and its metabolites were identified and quantified using GC‐ECD methods without confirmatory GC–MS or GC–MS/MS analysis due to the lack of instrumental facilities. Nevertheless, the analytical method demonstrated satisfactory validation performance, including acceptable linearity, recovery, precision, LOD, and LOQ, supporting the reliability of the quantitative determination of the target analytes. Future studies incorporating GC–MS or GC–MS/MS analysis would further strengthen the analytical reliability of pesticide residue determination in dried fish products.

## 4 Conclusion

This study presents a nationwide assessment of the occurrence of DDT and its metabolites in dried fish products. The analytical methods demonstrated robust performance, confirming their reliability for routine monitoring of pesticide residues in food samples. The contamination sequence among the targeted pesticides followed the pattern 4,4′‐DDE > 4,4′‐DDD > 4,4′‐DDT, suggesting the dominance of degradation products over the parent compound. This distribution is consistent with the persistence and environmental transformation of legacy DDT residues associated with historical application. However, the presence of parent 4,4′‐DDT in the absence of its degradation products, the ratio of 4,4′‐DDT/ΣDDTs approaching unity, and occasionally elevated concentrations in a limited number of samples are indicative of ongoing localized illegal application. The health risk assessment model revealed no significant non‐carcinogenic risk as the hazard quotient (HQ) was found to be well below the threshold of concern (HQ < 1) among the analyzed fish specimens. Similarly, the majority of incremental lifetime cancer risk (ILCR) fell within the negligible risk range (< 1 × 10^−6^). While a small number of samples exhibited elevated cumulative ILCR (ΣILCR), none exceeded the upper acceptable risk limit (1 × 10^−4^), indicating minimal carcinogenic risk associated with dried fish consumption. Although the estimated health risks at the consumption rate applied in this study (0.015 kg/person/day) were within acceptable limits, higher consumption rates, together with occasional elevated residue levels and the persistence and lipophilic nature of DDT, support the need for continued regulatory monitoring and control measures. The authorities must intensify the monitoring of fish‐drying practices and ensure strict application of the law against the use of banned pesticides in fishery products. Food regulatory bodies can play a vital role in training the fish processors and retailers on the safe and economical strategies of fish drying, processing, and packaging. Public awareness programs highlighting the adverse effects of using legacy pesticides such as DDT for food preservation should also be implemented. The study also recommends the continuous surveying of pesticide residues in other food‐chain items, including poultry and dairy products, to develop a comprehensive and updated national database on pesticide residues that will help to track the pesticide levels over time. This synergistic approach will be beneficial for the sustainable management of pesticide residue in the food chain, support policymakers in strengthening food safety regulations, and empower consumers to be aware of their dietary choices.

## Author Contributions

**Omeo Kumar Ghosh:** conceptualization, investigation, methodology, visualization, formal analysis, writing – original draft, writing – review and editing. **Md. Mazharul Islam:** conceptualization, investigation, methodology, visualization, writing – original draft, writing – review and editing. **Md. Iqbal Rouf Mamun:** conceptualization, investigation, methodology, visualization, supervision, writing – review and editing. **Md. Shaikat Hasan:** conceptualization, investigation, methodology, visualization, formal analysis. **Mohammad Shoeb:** conceptualization, investigation, methodology, visualization, funding acquisition, writing – review and editing. **Fajilatun Nesa:** conceptualization, investigation, methodology, visualization, formal analysis. **Syed Akhiar Ahsan Arham:** conceptualization, investigation, methodology, visualization, formal analysis. **Ashiqul Amin Abid:** conceptualization, investigation, methodology, visualization, formal analysis.

## Funding

The study was funded by the International Science Programme (ISP), Uppsala University, Sweden (Project No. BAN: 04).

## Ethics Statement

The authors have nothing to report.

## Conflicts of Interest

The authors declare no conflicts of interest.

## Supporting information


**Figure S1:** We would like to clarify that the calibration standards and analytical runs utilized a multi‐component standard mixture comprising a total of 20 Organochlorine Pesticides (OCPs). Consequently, additional peaks corresponding to these other OCP compounds naturally appear in the chromatograms. While a broad‐spectrum mixture was used for analytical tracking, the primary focus and scope of this research are specifically centered on DDT and its key metabolites (4,4′‐DDE, 4,4′‐DDD, and 4,4′‐DDT) in dried fish products. Therefore, quantification and reporting were purposefully restricted to these target analytes, while the other OCPs present in the mixture were omitted from the final results.
**Figure S2:** GC‐ECD chromatograms of blank.
**Figure S3:** GC‐ECD chromatograms of positive sample H‐23.
**Figure S4:** GC‐ECD chromatograms of positive sample H‐27.
**Figure S5:** GC‐ECD chromatograms of positive sample T‐16.

## Data Availability

The data that support the findings of this study are available from the corresponding author upon reasonable request.
