# Supplementary material for: DDT and Its Metabolites in Two Marine Dried Fish Species From Bangladesh: Residue Levels, Distribution Patterns, and Health Risk Assessment
Source: Food Sci Nutr. 2026 Sep 17;14(9):e72286. doi: 10.1002/fsn3.72286 (PMC13584756; doi:10.1002/fsn3.72286)
Supplement: Supplementary file 1 [file FSN3-14-e72286-s001.docx]

**Representative chromatograms of the standards, blank, and positive samples**

FIGURE S1 | GC-ECD chromatograms of standard 4,4´-DDE (RT: 23.21), 4,4´-DDD (RT: 25.46), and 4,4´-DDT (RT: 27.16)

**Clarification of Figure S1:** We would like to clarify that the calibration standards and analytical runs utilized a multi-component standard mixture comprising a total of 20 Organochlorine Pesticides (OCPs). Consequently, additional peaks corresponding to these other OCP compounds naturally appear in the chromatograms. While a broad-spectrum mixture was used for analytical tracking, the primary focus and scope of this research are specifically centered on DDT and its key metabolites (4,4'-DDE, 4,4'-DDD, and 4,4'-DDT) in dried fish products. Therefore, quantification and reporting were purposefully restricted to these target analytes, while the other OCPs present in the mixture were omitted from the final results.

FIGURE S2 | GC-ECD chromatograms of blank

FIGURE S3 | GC-ECD chromatograms of positive sample H-23

 FIGURE S4 | GC-ECD chromatograms of positive sample H-27

FIGURE S5 | GC-ECD chromatograms of positive sample T-16
